# Supplementary material for: Identification of Genes Involved in Fe–S Cluster Biosynthesis of Nitrogenase in Paenibacillus polymyxa WLY78
Source: Int J Mol Sci. 2021 Apr 5;22(7):3771. doi: 10.3390/ijms22073771 (PMC8038749; doi:10.3390/ijms22073771)
Supplement: Supplementary file 1 [file ijms-22-03771-s001.zip › Figures.docx]

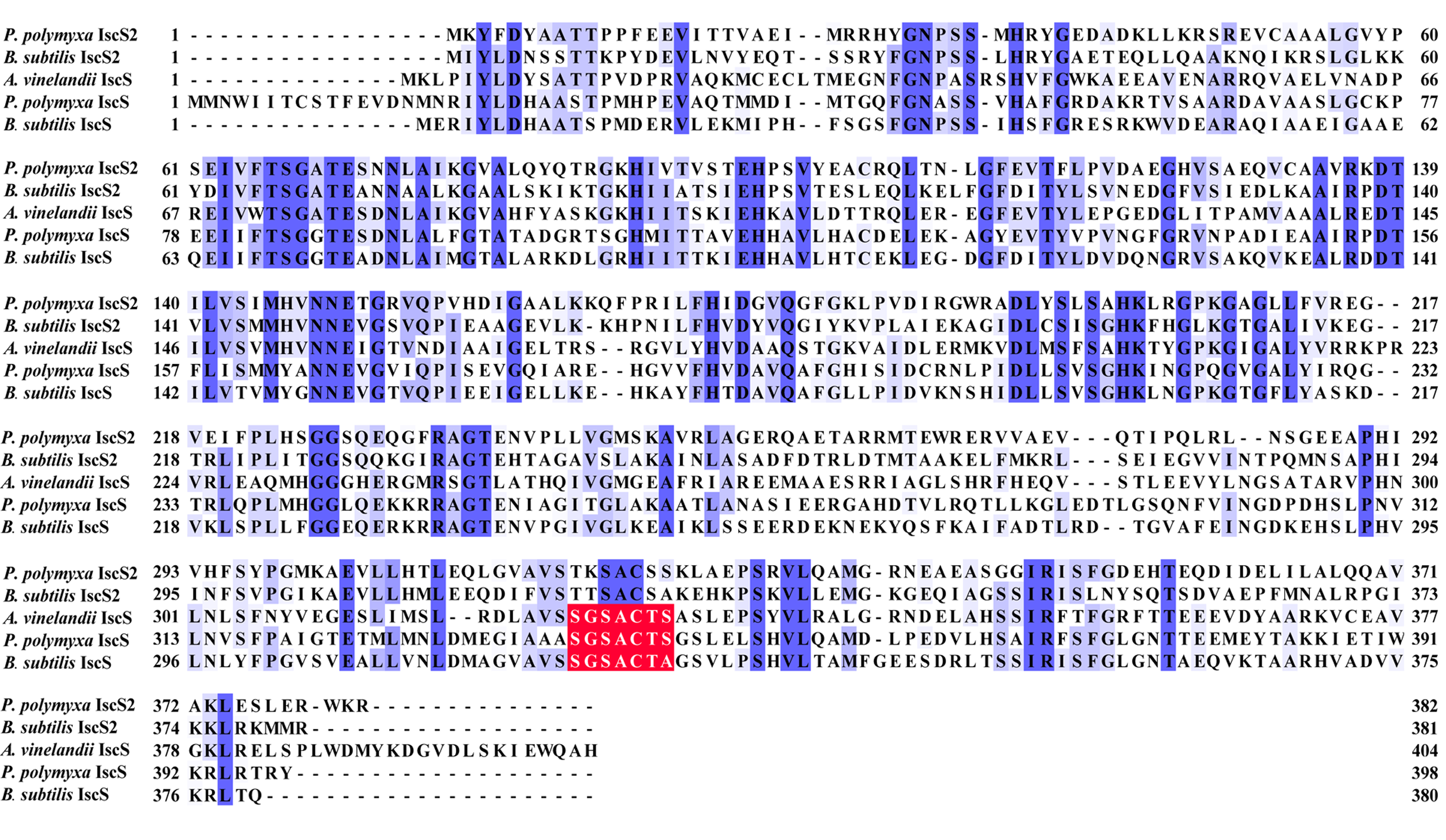


**Figure S1. Comparison of IscS and IscS2 from *P. polymyxa*, IscS from *A. vinelandii*, IscS and IscS2 from *B. subtilis*.** Protein sequences were aligned using the Clustal Omega program (<https://www.ebi.ac.uk/Tools/msa/clustalo/>, 23 February, 2020). Conserved SGSACTS motif are highlighted with a red background.


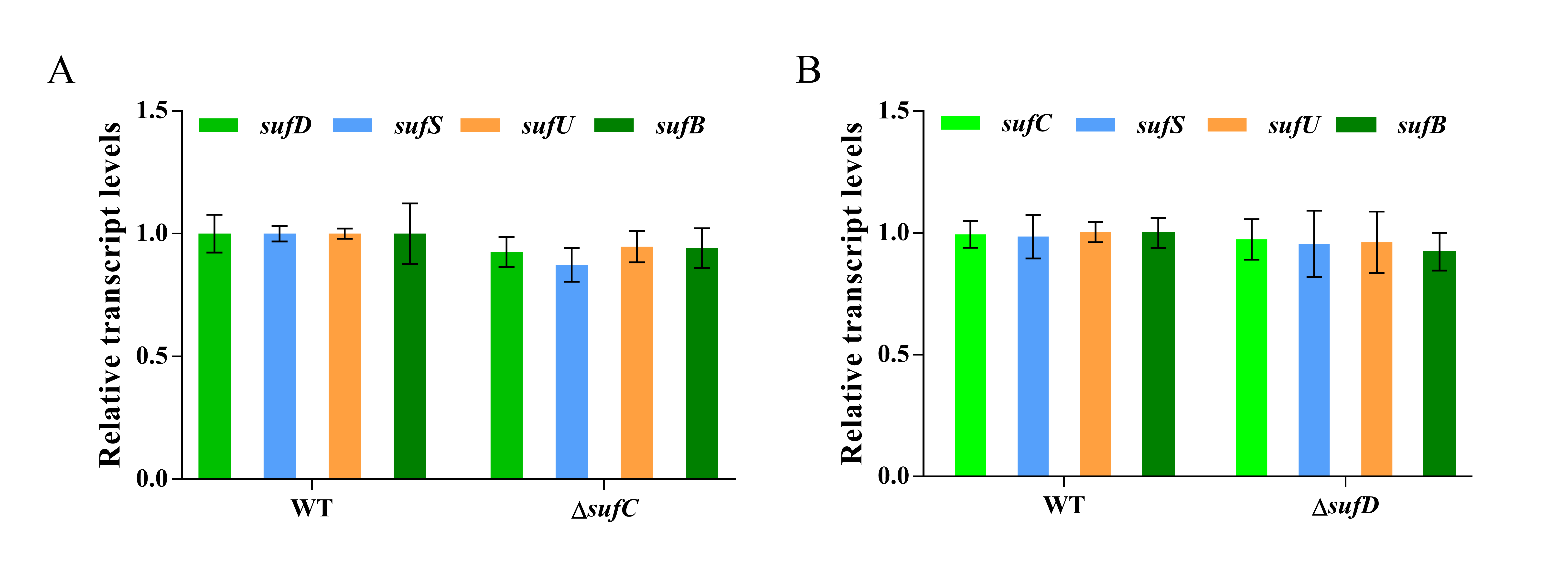


**Figure S2. The relative transcript levels of *suf* genes were determined by qRT-PCR.** (A) qRT-PCR analysis of the relative transcription levels of the *sufDSUB* genes in the WT and ∆*sufC* strains. **(B)** qRT-PCR analysis of the relative transcription levels of the *sufCSUB* genes in the WT and ∆*sufD* strains. The transcript level of the wild-type *P. polymyxa* WLY78 was used as a control. Results are representative of three independent experiments. Error bars indicate SD.


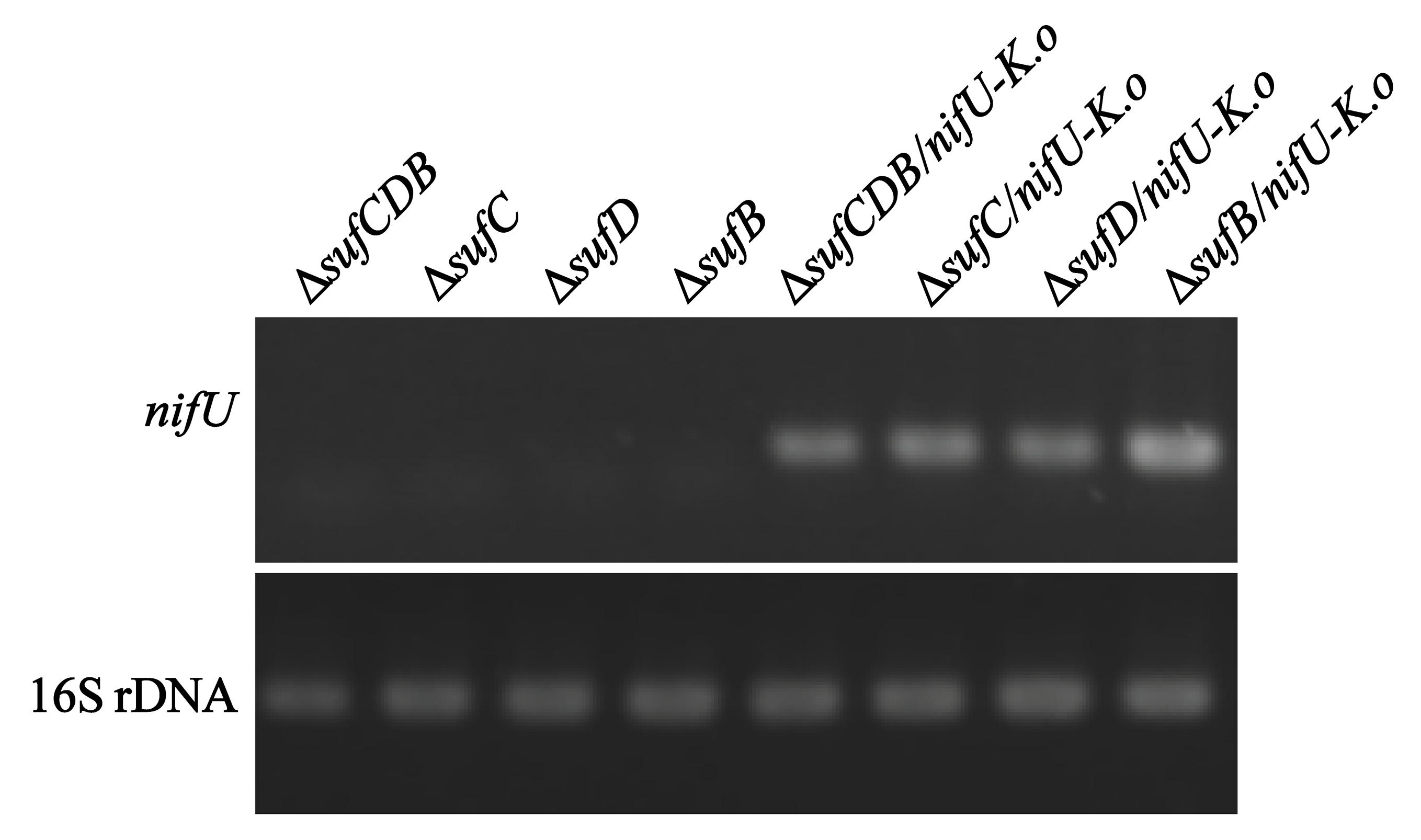


**Figure S3. RT-PCR analysis of *nifU* gene expression in Δ*sufCDB/nifU*-*K.o*, Δ*sufC*/*nifU*-*K.o*, Δ*sufD*/*nifU-K.o*, Δ*sufB*/*nifU-K.o* (complementation by *K. oxytoca nifU*) and WT (*P. polymyxa* WLY78).** RT-PCR reaction was performed to detect the level of 16S rDNA, to provide a loading control.


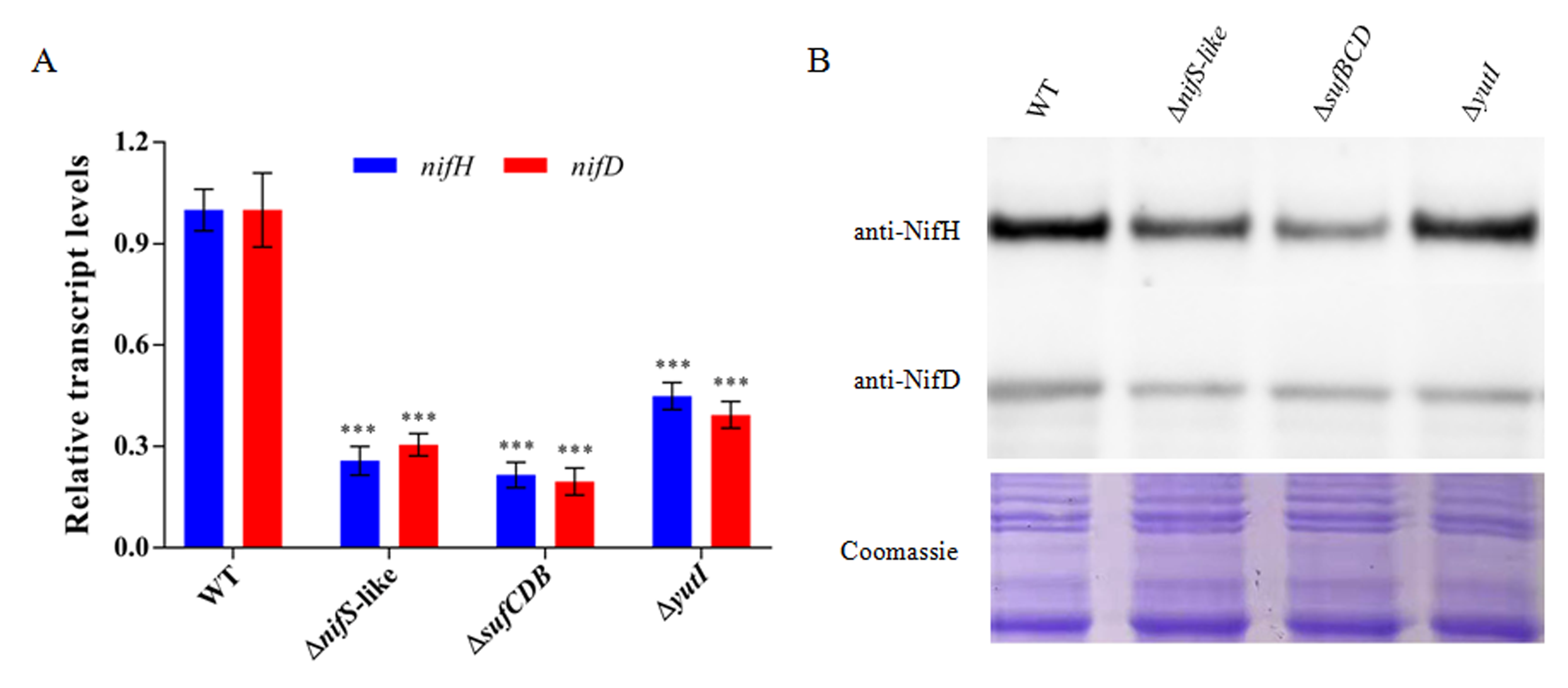


**Figure S4. Transcripts of *nifHD* genes and expression of NifHD proteins.** (A) qRT-PCR analysis of transcripts of *nifH* and *nifD* genes of ∆*nifS*-like, ∆*sufBCD*, ∆*yutI* and *P. polymyxa* WLY78. The transcription levels of *nifH* and *nifD* in the WT strain were arbitrarily set to 1.0. Results are representative of three independent experiments. Error bars indicate SD. ***P < 0.001 (B) Coomassie and western blot analysis of NifH and NifD expression in ∆*nifS*-like, ∆*sufCDB*, ∆*yutI* and *P. polymyxa* WLY78.
