# Supplementary material for: Identification of Genes Involved in Fe–S Cluster Biosynthesis of Nitrogenase in Paenibacillus polymyxa WLY78
Source: Int J Mol Sci. 2021 Apr 5;22(7):3771. doi: 10.3390/ijms22073771 (PMC8038749; doi:10.3390/ijms22073771)
Supplement: Supplementary file 1 [file ijms-22-03771-s001.zip › Table S1.docx]

**Table S1.** *P. polymyxa* WLY78 proteins predicted to contain Fe-S clusters.

| Gene id | Gene name | Protein | Proposed function/Name | Putative ligands | |
| --- | --- | --- | --- | --- | --- |
| GM000037 |  |  | arylsulfatase regulator | C109-C113-C116-C397-C400-C406 | |
| GM000086 | *oppD* | OppD | peptide ABC transporter ATP-binding protein | C299-C305-C312-C329 | |
| GM000185 |  |  | hypothetical protein | C232-C235-C241 | |
| GM000242 |  |  | radical SAM protein | C186-C190-C193 | |
| GM000397 | *parA* | ParA | sporulation initiation inhibitor |  | |
| GM000411 | *appD* | AppD | peptide ABC transporter ATPase | C285-C291-C298-C316 | |
| GM000412 | *appF* | AppF | peptide ABC transporter substrate-binding protein | C287-C293-C300-C318 | |
| GM000515 | *phnA* | PhnA | hypothetical protein |  | |
| GM000518 |  |  | Fe-S oxidoreductase | C31-C35-C38-C271 | |
| GM000532 | *yhfW* | YhfW | (2Fe-2S)-binding protein | C466-H468-C484-H487 | |
| GM000549 | *rlmN* | RlmN | RNA methyltransferase | C112-C116-C119 | |
| GM000550 | *pyrK* | PyrK | diguanylate cyclase | C194-C199-C202-C214 | |
| GM000631 |  |  | hypothetical protein | C13 | |
| GM000787 | *nrdG* | NrdG | ribonucleoside-triphosphate reductase activating protein | C26-C30-C33 | |
| GM001024 | *nirA* | NirA | ferredoxin--nitrite reductase | C396-C402-C437-C441 | |
| GM001118 | *rpoA* | RpoA | DNA-directed RNA polymerase alpha chain |  | |
| GM001124 | *cysH* | CysH | phosphoadenosine phosphosulfate reductase | C117-C118-C200-C203 | |
| GM001127 | *salA* | SalA | ATP-binding protein |  | |
| GM001283 |  |  | glutaredoxin | C12 | |
| GM001364 | ***sufD2*** | **SufD2** | **Fe-S cluster assembly protein** |  | |
| GM001365 | ***sufB2*** | **SufB2** | **Fe-S cluster assembly protein** |  | |
| GM001367 | ***nfuA*** | **NfuA** | **nitrogen-fixing protein NifU** |  | |
| GM001407 |  |  | DNA photolyase | C33-C37-C40 | |
| GM001415 | *lipA* | LipA | lipoyl synthase | C41-C46-C52-C67-C71-C74 | |
| GM001452 | ***yutI*** | **YutI** | **nitrogen-fixing protein NifU** |  | |
| GM001455 | *mqnE* | MqnE | radical SAM protein | C61-C65-C68 | |
| GM001456 | ***sufA*** | **SufA** | **iscA iron-sulfur cluster assembly protein** | C115-C117 | |
| GM001483 | *trxA* | TrxA | thioredoxin | C30 | |
| GM001491 | *sdhB* | SdhB | Succinate dehydrogenase iron-sulfur subunit | C69-C74-C77-C89-C160-C163-C166-C170-C217-C223-C227 | |
| GM001578 |  |  | radical SAM protein | C53-C57-C60 | |
| GM001672 | *ispG* | IspG | 4-hydroxy-3-methylbut-2-en-1-yl diphosphate synthase | C265-C268-C300-E307 | |
| GM001698 | *minD* | MinD | Septum site-determining protein |  | |
| GM001771 | ***iscS*** | **IscS** | **cysteine desulfurase** | C342 | |
| GM001962 | *parA* | ParA | hypothetical protein |  | |
| GM002012 |  |  | Fe-S oxidoreductase | C25-C29-C32-C324-C327 | |
| GM002017 | *narG* | NarG | Nitrate reductase alpha chain | H48-C52-C56 | |
| GM002018 | *narH* | NarH | Nitrate reductase | C183-C186-C191-C195-C216-C222-C226-C243-C246-C258-C262 | |
| GM002029 | *moaD* | MoaD | molybdopterin converting factor | C25-C29-C32-C262-C265-C279 | |
| GM002089 |  |  | ferric iron reductase |  | |
| GM002137 |  |  | oxidoreductase | C38-C49-C52-C64-H96-C100-C103-C110-C151-C154-C157-C161-C194-C197-C200-C204-C206-C268-C271-C275-C303 | |
| GM002138 | *moaA* | MoaA | molybdenum cofactor biosynthesis protein A | C230-C233-C247 | |
| GM002206 | *hemN* | HemN | coproporphyrinogen III oxidase | C24-C28-C31 | |
| GM002223 |  |  | 30S ribosomal protein S12 methylthiotransferase | C21-C58-C89-C164-C168-C171 | |
| GM002227 | *addB* | AddB | ATP-dependent helicase | C1141-C1144-C1150 | |
| GM002268 | ***iscS2*** | **IscS2** | **cysteine desulfurase** | C322 | |
| GM002404 | *nasD* | NasD | nitrite reductase | C413-C415-C448-C451 | |
| GM002405 | *nasC* | NasC | nitrite reductase | C47-C50-C54-C88 | |
| GM002408 | *nasD* | NasD | nitrite reductase | C422-C424-C456-C459-C484-C486-C521-C524-C637-C643-C677-C681 | |
| GM002409 | *nasE* | NasE | Assimilatory nitrite reductase [NAD(P)H] small subunit | C50-H52-C69-H72 | |
| GM002473 | *rlmN* | RlmN | Ribosomal RNA large subunit methyltransferase N | C120-C124-C127 | |
| GM002512 |  |  | pyruvate formate lyase-activating protein | C29-C33-C36 | |
| GM002520 | *splB* | SplB | radical SAM protein | C103-C107-C110 | |
| GM002592 | *porG* | PorG | ferredoxin | C256-C259-C295-C298-C301 | |
| GM002629 | *fdx* | Fdx | ferredoxin | C37-C43-C46-C81 | |
| GM002710 | *bioB* | BioB | biotin synthase | C46-C50-C53-C296-C304-C307 | |
| GM002711 | *thiH* | ThiH | thiamine biosynthesis protein | C96-C100-C103 | |
| GM002714 | *hydN* | HydN | electron transporter HydN | C12-C15-C18-C59-C62-C67-C71-C90-C93-C96-C100-C141-C144-C153 | |
| GM002715 | *nuoG* | NuoG | iron hydrogenase | C18-C21-C24-C28-D31-C48-C51-C54-C58-C192-C247-C391-C395 | |
| GM002716 | *hydN* | HydN | Electron transport protein HydN | C43-C46-C49-C92-C95-C100-C104-C123-C126-C129-C133-C185 | |
| GM002717 | *fdhF* | FdhF | formate dehydrogenase | C9-C12-C16 | |
| GM002736 | *leuC* | LeuC | isopropylmalate isomerase | C342 | |
| GM002741 | *nth* | Nth | endonuclease III | C188-C195-C198-C204 | |
| GM002771 | *hemN* | HemN | coproporphyrinogen III oxidase | C89-C93-C96 | |
| GM002954 |  |  | hypothetical protein | C104-C108-C111 | |
| GM003122 | *gltB* | GltB | glutamate synthase | C51-C54-C59-C72 | |
| GM003188 | *bioB* | BioB | biotin synthase | C71-C75-C78 | |
| GM003364 | *narH* | NarH | Nitrate reductase | C144-C152-C156-C177-C183-C187-C204-C207-C219 | |
| GM003365 | *narG* | NarG | Nitrate reductase alpha chain | H53-C57-C61 | |
| GM003546 |  |  | 2, 4-dienoyl-CoA reductase | C328-C331-C335-C348 | |
| GM003585 | *nadA* | NadA | quinolinate synthetase | C230 | |
| GM003586 | ***nifS-*like** | **NifS-like** | **cysteine desulfurase** | C338 | |
| GM003618 | *lutB* | LutB | lactate utilization protein B | C316-C319-C322-C326-C370-C373-C377;;C316-C319-C322 | |
| GM003619 | *lutA* | LutA | Fe-S oxidoreductase | CCG | |
| GM003681 |  |  | peptide ABC transporter substrate-binding protein | C287-C293-C300-C318 | |
| GM003682 | *appD* | AppD | peptide ABC transporter ATPase | C286-C292-C299-C317 | |
| GM003698 | *ytnI* | YtnI | glutaredoxin | C17 | |
| GM003759 | *cbiW* | CbiW | ferredoxin | C22-C27-C59-C63 | |
| GM003826 | *miaB* | MiaB | dimethylallyladenosine tRNA methylthiotransferase | C83-C228-C232-C235 | |
| GM003891 | *flhG* | FlhG | cobyrinic acid a, c-diamide synthase |  | |
| GM004082 | *fer* | Fer | Ferredoxin | C12-C15-C18 | |
| GM004184 |  |  | Fe-S oxidoreductase | C16-C20-C23-C219-C237 | |
| GM004217 | *accD* | AccD | acetyl-CoA carboxylase subunit beta | C44-C47-C63-C66 | |
| GM004240 | *yfkA* | YfkA | Putative protein YfkA | C44-C48-C51 | |
| GM004247 | ***sufB*** | **SufB** | **Fe-S cluster assembly protein** |  | |
| GM004248 | ***sufU*** | **SufU** | **Zinc-dependent sulfurtransferase SufU** | C124 | |
| GM004249 | ***sufS*** | **SufS** | **cysteine desulfurase** | C362 | |
| GM004250 | ***sufD*** | **SufD** | **Fe-S cluster assembly protein** |  | |
| GM004315 |  |  | ciliary or flagellar motility | C90-C94-C97 | |
| GM004345 | *ispH* | IspH | 4-hydroxy-3-methylbut-2-enyl  diphosphate reductase | | |
| GM004356 | *mqnC* | MqnC | radical SAM protein | | C65-C69-C72 |
| GM004407 | *ytqA* | YtqA | hypothetical protein | | C55-C58 |
| GM004469 | *bchE* | BchE | Fe-S oxidoreductase | | C186-C190-C193 |
| GM004562 |  |  | CRISPR-associated protein Cas4 | | C86-C89-C95 |
| GM004726 | *ywqD* | YwqD | capsular biosynthesis protein | |  |
| GM004745 |  |  | tyrosine protein kinase | |  |
| GM004750 | *gltB* | GltB | Ferredoxin-dependent glutamate synthase | | C1132-C1138-C1143 |
| GM004796 | *queE* | QueE | 7-carboxy-7-deazaguanine synthase | | C41-C45-C48 |
| GM004817 | *hcp* | Hcp | hydroxylamine reductase | | H110-E134-C178-C264-C292-C319-E353 |
| GM004855 |  |  | hypothetical protein | | C20-C291-C294-C300 |
| GM004891 | ***nifN*** | **NifN** | **nitrogenase iron-molybdenum cofactor biosynthesis protein** | | C43 |
| GM004892 | ***nifE*** | **NifE** | **nitrogenase iron-molybdenum cofactor biosynthesis protein** | | C36-C61-C122 |
| GM004893 | ***nifK*** | **NifK** | **nitrogenase molybdenum-iron protein subunit beta** | | C93-C151 |
| GM004894 | ***nifD*** | **NifD** | **nitrogenase molybdenum-iron protein subunit alpha** | | C62-C88-C154 |
| GM004895 | ***nifH*** | **NifH** | **nitrogenase reductase** | | C97-C131 |
| GM004896 | ***nifB*** | **NifB** | **nitrogen fixation protein NifB** | | C42-C46-C49 |
| GM004903 |  |  | hypothetical protein | | C22-C24-C57-C62 |
| GM004941 |  |  | hypothetical protein | | C219-C222-C228 |
| GM004944 |  |  | hypothetical protein | | C295-C298 |
| GM004949 |  |  | hypothetical protein | | C14-C18-C21 |
| GM005077 |  |  | RNA methyltransferase | | C176-C182-C185-C309 |
| GM005131 | *purF* | PurF | amidophosphoribosyltransferase | | C413-C469-C472 |
| GM005189 | *acoA* | AcoA | aconitate hydratase | | C495 |
| GM005201 | *queG* | QueG | Epoxyqueuosine reductase | | C201-C204-C207-C211-C227-C253-C256 |
| GM005216 | *yfhQ* | YfhQ | adenine glycosylase | | C197-C204-C207-C213 |
| GM005286 |  |  | Fe-S oxidoreductase | | C11-C15-C18-C239-C257-C295-C298 |
| GM005287 |  |  | Fe-S oxidoreductase | | C350-C353-C359 |
| GM005309 | *nasD* | NasD | nitrite reductase (NAD(P)H) large subunit | | C421-C423-C456-C459-C484-C486-C521-C524-C637-C643-C677-C681 |
| GM005639 | *sdaAA* | SdaAA | serine dehydratase | | C168-C210-C221 |
