# Supplementary material for: Identification of Genes Involved in Fe–S Cluster Biosynthesis of Nitrogenase in Paenibacillus polymyxa WLY78
Source: Int J Mol Sci. 2021 Apr 5;22(7):3771. doi: 10.3390/ijms22073771 (PMC8038749; doi:10.3390/ijms22073771)
Supplement: Supplementary file 1 [file ijms-22-03771-s001.zip › Table S2.docx]

**Table S2.** Bacterial strains and plasmids used in this study.

| **Strain or plasmid** | **Genotype and/or relevant characteristics** | **Source or reference** |
| --- | --- | --- |
| **strains** | | |
| *Paenibacillus polymyxa* |  |  |
| WLY78 | Wild-type strain | Laboratory stock |
| Δ*sufC* | *sufC* in-frame deletion mutant | This study |
| Δ*sufD* | *sufD* in-frame deletion mutant | This study |
| Δ*sufB* | *sufB* in-frame deletion mutant | This study |
| Δ*sufA* | *sufA* in-frame deletion mutant | This study |
| Δ*sufCDB* | *sufC, sufD* and *sufB* triple deletion mutant | This study |
| Δ*sufC2D2B2* | *sufC2D2B2* in-frame deletion mutant | This study |
| Δ*iscS* | *iscS1* in-frame deletion mutant | This study |
| Δ*iscS2* | *iscS2* in-frame deletion mutant | This study |
| Δ*iscS/*Δ*iscS2* | *iscS1* and *iscS2* double deletion mutant | This study |
| Δ*nifS*-like | *nifS*-like in-frame deletion mutant | This study |
| Δ*nfuA*-like | *nfuA-*like in-frame deletion mutant | This study |
| Δ*yutI* | *yutI* in-frame deletion mutant | This study |
| Δ*nfuA*-like*/*Δ*yutI* | *nfuA*-like and *yutI* double deletion mutant | This study |
| Δ*sufC*/*sufC* | Complementation strain of Δ*sufC* with *sufC* carried in plasmid pHYsufC | This study |
| Δ*sufD*/*sufD* | Complementation strain of Δ*sufD* with *sufD* carried in plasmid pHYsufD | This study |
| Δ*sufB*/*sufB* | Complementation strain of Δ*sufB* with *sufB* carried in plasmid pHYsufB | This study |
| Δ*sufCDB*/*sufCDB* | Complementation strain of Δ*sufCDB* with *sufCD* and *sufB* carried in plasmid pHYsufCDB | This study |
| Δ*sufCDB/nifU-K. o* | Complementation strain of Δ*sufCDB* with *nifU* of *K. oxytoca* carried in plasmid pHYnifU | This study |
| Δ*sufC/nifU-K. o* | Complementation strain of Δ*sufC* with *nifU* of *K. oxytoca* carried in plasmid pHYnifU | This study |
| Δ*sufD/nifU-K. o* | Complementation strain of Δ*sufD* with *nifU* of *K. oxytoca* carried in plasmid pHYnifU | This study |
| Δ*sufB/nifU-K. o* | Complementation strain of Δ*sufB* with *nifU* of *K. oxytoca* carried in plasmid pHYnifU | This study |
| Δ*nifS*-like/*nifS*-like | Complementation strain of Δ*nifS*-like with *nifS*-like carried in plasmid pHYnifS | This study |
| Δ*nifS*-like/*nifS -K. o* | Complementation strain of Δ*nifS*-like with *nifS* of *K. oxytoca* carried in plasmid pHYnifS (*K. o*) | This study |
| Δ*yutI*/*yutI* | Complementation strain of Δ*yutI* with *yutI* carried in plasmid pHYyutI | This study |
| Δ*yutI/yutI-*49 | Complementation strain of Δ*yutI* with cysteine to alanine substitutions at residues 49 of YutI | This study |
| Δ*yutI/yutI-*52 | Complementation strain of Δ*yutI* with cysteine to alanine substitutions at residues 52 of YutI | This study |
| Δ*yutI/yutI-*4952 | Complementation strain of Δ*yutI* with cysteine to alanine substitutions at residues 49 and 52 of YutI | This study |
| Δ*yutI*/*nifU -K. o* | Complementation strain of Δ*yutI* with *nifU* of *K. oxytoca* carried in plasmid pHYnifU (*K. o*) | This study |
| Δ*yutI*/*nfuA-E. coli* | Complementation strain of Δ*yutI* with *nfuA* of *E. coli* carried in plasmid pHYnfuA (*E. coli*) | This study |
| *E. coli* | | |
| JM109 | General cloning host*; recA1, endA1, gyrA96, thi-1, hsdR17, supE44, relA1, Δ(lac-proAB*)/F’[*traD36, proAB+, lacIq, lacZ*ΔM15] | Sangon Biotech Co. |
| **Plasmids** | | |
| pHY300PLK | Multiple-copy *E. coli-Bacillus* shuttle vector, Tc^r^ | TaKaRa |
| pRN5101 | Temperature-sensitive *E. coli-Bacillus* shuttle vector, Em^r^ | 48 |
| pRDsufC | *sufC* deletion vector based on pRN5101 | This study |
| pRDsufD | *sufD* deletion vector based on pRN5101 | This study |
| pRDsufB | *sufB* deletion vector based on pRN5101 | This study |
| pRDsufCD | *sufCD* deletion vector based on pRN5101 | This study |
| pRDsufC2D2B2 | *sufC2D2B2* deletion vector based on pRN5101 | This study |
| pRDsufA | *sufA* deletion vector based on pRN5101 | This study |
| pRDiscS | *iscS* deletion vector based on pRN5101 | This study |
| pRDiscS2 | *iscS2* deletion vector based on pRN5101 | This study |
| pRDnifS | *nifS*-like deletion vector based on pRN5101 | This study |
| pRDnfuA | *nfuA*-like deletion vector based on pRN5101 | This study |
| pRDyutI | *yutI* deletion vector based on pRN5101 | This study |
| pHYsuf | *sufCDB* complemented vector with *sufCDB* in pHY300PLK | This study |
| pHYnifS | *nifS*-like complemented vector with *nifS*-like in pHY300PLK | This study |
| pHYyutI | *yutI* complemented vector with *yutI* in pHY300PLK | This study |
| pHYnifU (K. o) | *sufCDB* complemented vector with *nifU* of *K. oxytoca* in pHY300PLK | This study |
| pHYnifS (K. o) | *nifS*-like complemented vector with *nifS of K. oxytoca* in pHY300PLK | This study |
| pHYnfuA (E. coli) | *yutI* complemented vector with *nfuA of E. coli* in pHY300PLK | This study |
| pHYyutI-C49A | *yutI* complemented vector with cysteine to alanine substitutions at residues 49 of YutI in pHY300PLK | This study |
| pHYyutI-C52A | *yutI* complemented vector with cysteine to alanine substitutions at residues 52 of YutI in pHY300PLK | This study |
| pHYyutI-C49/52A | *yutI* complemented vector with cysteine to alanine substitutions at residues 49 and 52 of YutI in pHY300PLK | This study |
