# Supplementary material for: Identification of Genes Involved in Fe–S Cluster Biosynthesis of Nitrogenase in Paenibacillus polymyxa WLY78
Source: Int J Mol Sci. 2021 Apr 5;22(7):3771. doi: 10.3390/ijms22073771 (PMC8038749; doi:10.3390/ijms22073771)
Supplement: Supplementary file 1 [file ijms-22-03771-s001.zip › Table S3.docx]

**Table S3** Primers used in this study.

| **Primer name** | **Sequence** | **Location /Target** |
| --- | --- | --- |
| sufB-U-F | CGGCCACGATGCGTCCGGCGTAGAGATAATGCCAAAATACCAGC | Upstream of *sufB* |
| sufB-U-R | AGGTTTGAGAAACAATCCATTAACCCTTGGTG |  |
| sufB-D-F | GGGTTAATGGATTGTTTCTCAAACCTCCTCTG | Downstream of *sufB* |
| sufB-D-R | GACTGCGCAAAAGACATAATCGATACACAAAATGTGTGCGCCTAC |  |
| sufD-U-F | CGGCCACGATGCGTCCGGCGTAGAGCGTTCTGCCGGTCCGTATATC | Upstream of *sufD* |
| sufD-U-R | GCGAGGAGGAAAACTAGAAAGGAAGTTAGGCCAATGAATAC |  |
| sufD-D-F | CTAACTTCCTTTCTAGTTTTCCTCCTCGCTTC | Downstream of *sufD* |
| sufD-D-R | GACTGCGCAAAAGACATAATCGATAAAGTTGGATAACGTGTGTC |  |
| sufC-U-F | CGGCCACGATGCGTCCGGCGTAGAGATGGAAGACGCTTCTTCACGC | Upstream of *sufC* |
| sufC-U-R | CACACTACATTTTGCCGGAAGCGAGGAGGAA |  |
| sufC-D-F | CCTCGCTTCCGGCAAAATGTAGTGTGGTGTC | Downstream of *sufC* |
| sufC-D-R | GACTGCGCAAAAGACATAATCGATACTTCTTTTTGTTTCGTTTCC |  |
| sufD-U-F | CGGCCACGATGCGTCCGGCGTAGAGCGTTCTGCCGGTCCGTATATC | Upstream of *sufCD* |
| sufCD-U-R | CACACTACATTTTTTGAAAGGAAGTTAGGCCAATGAATAC |  |
| sufCD-D-F | ACTTCCTTTCAAAAAATGTAGTGTGGTGTCGAAATCAG | Downstream of *sufCD* |
| sufC-D-R | GACTGCGCAAAAGACATAATCGATACTTCTTTTTGTTTCGTTTCC |  |
| sufA-U-F | CGGCCACGATGCGTCCGGCGTAGAGGAATTTGCAGCATATGTTCA | Upstream of *sufA* |
| sufA-U-R | CGTTCGGTTT GTTCTCCTTCAGTATACTCCAC |  |
| sufA-D-F | GAAGGAGAAC AAACCGAACG AAGAGCCTTG TT | Downstream of *sufA* |
| sufA-D-R | GACTGCGCAAAAGACATAATCGATATGACTTCGACCATGTAGCCAT |  |
| sufC2D2B2-U-F | CGGCCACGATGCGTCCGGCGTAGAGATA ACATATCCAGACACAGG | Upstream of *sufC2D2B2* |
| sufC2D2B2-U-R | CTATTAAATTAATGAATTATCGTAAACTTCCCTTC |  |
| sufC2D2B2-D-F | AAGTTTACGATAATTCATTAATTTAATAGATCAAACCTTTATC | Downstream of *sufC2D2B2* |
| sufC2D2B2-D-R | GACTGCGCAAAAGACATAATCGATACTAATAGCGAGTCTTTTCGCA |  |
| nifS-U- F | CGGCCACGATGCGTCCGGCGTAGAGCATTGGGTCCCTGGCTCTG | Upstream of *nifS*-like |
| nifS-U-R | TTTAAAGATTCAGTCAAAAACTCTTGAGATTATTGTAGGCATG |  |
| nifS-D-F | CAAGAGTTTTTGACTGAATCTTTAAAACGGTCAGG | Downstream of *nifS*-like |
| nifS-D- R | GACTGCGCAAAAGACATAATCGATACACTCAGCCAAGGACGAC |  |
| nfuA-U-F | CGGCCACGATGCGTCCGGCGTAGAGTATTTATAGTACCAGTTCTCTGC | Upstream of *nfuA*-like |
| nfuA-U-R | AGTATCAAGGAATTGTTTTACCAACCGTCAATTC |  |
| nfuA-D-F | GGTTGGTAAAACAATTCCTTGATACTGAGCAAC | Downstream of *nfuA*-like |
| nfuA-D-R | GACTGCGCAAAAGACATAATCGATATTTTCTTAGTTTTCATATAAAAAAGTG |  |
| yutI-U-F | CGGCCACGATGCGTCCGGCGTAGAGAGTAGGGAACGAGAACAC | Upstream of *yutI* |
| yutI-U-R | GAGTTGAATTCTATGCTTGATGCATAATGATC |  |
| yutI-D-F | ATGCATCAAGCATAGAATTCAACTCCTTTCC | Downstream of *yutI* |
| yutI-D-R | GACTGCGCAAAAGACATAATCGATATTTGAATATTACCATTATGGATCG |  |
| P-sufC-F | ATGGAAAAACGCTTTGCCCATTGTAGTGATGGGATTACG | Promoter region of *sufCDSUB* operon and *sufC* ORF |
| C-sufC-R | TTATAACAGGAATTCCCGGGTAAGCCGGAAGCGAGGAGGA |  |
| P-sufC-F | ATGGAAAAACGCTTTGCCCATTGTAGTGATGGGATTACG | Promoter region of *sufCDSUB* operon |
| P-suf-R (sufD) | TGTGTTGTCATATTCGCAATACCCTCCAAAAAAG |  |
| C-sufD-F | GGTATTGCGAATATGACAACACAAACAATCCT | *sufD* ORF |
| C-sufD-R | TTATAACAGGAATTCCCGGGCGGGAATTGTTCACGGATCA |  |
| P-sufC-F | ATGGAAAAACGCTTTGCCCATTGTAGTGATGGGATTACG | Promoter region of *sufCDSUB* operon |
| P-suf-R (sufB) | GCTTTCTTGGCCATATTCGCAATACCCTCCAAAAAATG |  |
| C-sufB-F | GGTATTGCGAATATGGCCAAGAAAGCACCGGA | *sufB* ORF |
| C-sufB-R | TTATAACAGGAATTCCCGGGTGGATTTTAACCAATACTGC |  |
| P-sufC-F | ATGGAAAAACGCTTTGCCCATTGTAGTGATGGGATTACG | Construction of pHYsufCDB for complementation of ∆*sufCDB* |
| C-sufCD-R | CTGTTCTATTTAAATCATTGGCCTAACTTCCTTTC |  |
| C-sufCD-F | GTTAGGCCAATGATTTAAATAGAACAGAGGAGG |  |
| C-sufB-R | TTATAACAGGAATTCCCGGGTGGATTTTAACCAATACTGC |  |
| P-yutI-F | ATGGAAAAACGCTTTGCCCAACAGAGCATTATATTTCAATCC | Promoter region of *yutI* gene |
| P-yutI-R | AATTCCACATTAGAATTCAACTCCTTTCC |  |
| C-nifU-F | TTGAATTCTAATGTGGAATTACTCCGAGAAAGTGAAAG | *nifU* ORF of *K. oxytoca* and RT-PCR for *nifU* |
| C-nifU-R | TTATAACAGGAATTCCCGGGTCAGGCCGCCACCACTTC |  |
| C-nifS-F | ATGGAAAAACGCTTTGCCCATCCGCTTCCTGATTTTGTG | *nifS*-like and its promoter |
| C-nifS-R | TTATAACAGGAATTCCCGGGCCTGACCGTTTTAAAGATTC |  |
| P-nifS-F | ATGGAAAAACGCTTTGCCCATCCGCTTCCTGATTTTGTG | Promoter region of *nifS*-like gene |
| P-nifS-R | CCTGTTTCATAAACTCTTGAGATTATTGTAGGC |  |
| C-nifS-F-K. o | TCAAGAGTTTATGAAACAGGTTTATCTCGATAACAACG | *nifS* ORF of *K. oxytoca* |
| C-nifS-R-K. o | TTATAACAGGAATTCCCGGGTTAGCCGTAAACCGGCGTG |  |
| P-yutI-F | ATGGAAAAACGCTTTGCCCAACAGAGCATTATATTTCAATCC | *yutI* gene and its promoter |
| C-yutI-R | TTATAACAGGAATTCCCGGGGATCATTATGCATCAAGCATTAAAATAC |  |
| P-yutI-F | ATGGAAAAACGCTTTGCCCAACAGAGCATTATATTTCAATCC | Construction of YutIC49A variant for complementation of ∆*yutI* |
| YutIC49A-R | ACTGCCTGCAGCGCCTACCAGCTTCAG |  |
| YutIC49A-F | GTAGGCGCTGCAGGCAGTTGCCCAAGTTCC |  |
| C-yutI-R | TTATAACAGGAATTCCCGGGGATCATTATGCATCAAGCATTAAAATAC |  |
| P-yutI-F | ATGGAAAAACGCTTTGCCCAACAGAGCATTATATTTCAATCC | Construction of YutIC52A variant for complementation of  ∆*yutI* |
| YutIC52A-R | ACTTGGTGCACTGCCGCAAGCGCCTAC |  |
| YutIC52A-F | TGCGGCAGTGCACCAAGTTCCACAATCACG |  |
| C-yutI-R | TTATAACAGGAATTCCCGGGGATCATTATGCATCAAGCATTAAAATAC |  |
| P-yutI-F | ATGGAAAAACGCTTTGCCCAACAGAGCATTATATTTCAATCC | Construction of YutIC49/52A variant for complementation of ∆*yutI* |
| YutIC49/52A-R | TGGTGCACTGCCTGCAGCGCCTACCAGCTTCAGCT |  |
| YutIC49/52A-F | GTAGGCGCTGCAGGCAGTGCACCAAGTTCCACAATCACG |  |
| C-yutI-R | TTATAACAGGAATTCCCGGGGATCATTATGCATCAAGCATTAAAATAC |  |
| P-yutI-F | ATGGAAAAACGCTTTGCCCAACAGAGCATTATATTTCAATCC | Promoter region of *yutI* gene |
| P-yutI-R (nfuA) | TACGGATCATTAGAATTCAACTCCTTTCC |  |
| C-nfuA-F | TTGAATTCTAATGATCCGTATTTCCGATG | *nfuA* ORF of *E. coli* |
| C-nfuA-R | TTATAACAGGAATTCCCGGGAAAAGAGGGGATAACTTAGTAG |  |
| nifHF | ACCTGCCAGCTCTTCATACTC | qRT-PCR for *nifH* |
| nifHR | AACAGCCGGAATACGGACC |  |
| nifDF | TCATTCCTGTACGCTGTGAGG | qRT-PCR for *nifD* |
| nifDR | CACCGCCGATATTGTAGTCTC |  |
| sufCF | CGTCTGCTTTGATGGGTCA | qRT-PCR for *sufC* |
| sufCR | GATTTCTTGTCCTTCTTCACGG |  |
| sufDF | GGTGCGGTAGAAGTTGTTGC | qRT-PCR for *sufD* |
| sufDR | GCTTGCTGTATTGCCGTTG |  |
| sufSF | ATCTGATTCCGTGGCAACA | qRT-PCR for *sufS* |
| sufSR | TTGACAGGGTTCACAACACC |  |
| sufUF | AAACGAAAGACGGCGTGGT | qRT-PCR for *sufU* |
| sufUR | AGCCAGTGTCGCACATTTG |  |
| sufBF | TAAACTGGGTATTCCCGAGGC | qRT-PCR for *sufB* |
| sufBR | TCTCAGGATGCTCACGCAA |  |
| 16S-F | TTTGTCGTCAGCCTCGTGTTCGTG | qRT-PCR for control (16S rDNA) |
| 16S-R | ATCCCCACCTTCCTCCGGTTTG |  |
